# Determination of Vitamin B3 Vitamer (Nicotinamide) and Vitamin B6 Vitamers in Human Hair Using LC-MS/MS

**DOI:** 10.3390/molecules26154487

**Published:** 2021-07-25

**Authors:** Sundus M. Sallabi, Aishah Alhmoudi, Manal Alshekaili, Iltaf Shah

**Affiliations:** Department of Chemistry, College of Science, UAE University, Al Ain 15551, United Arab Emirates; 201640052@uaeu.ac.ae (S.M.S.); 201303508@uaeu.ac.ae (A.A.); 201250724@uaeu.ac.ae (M.A.)

**Keywords:** nicotinamide, vitamin B3, vitamin B6, hair analysis, vitamin, vitamers, LC-MS/MS

## Abstract

Water-soluble B vitamins participate in numerous crucial metabolic reactions and are critical for maintaining our health. Vitamin B deficiencies cause many different types of diseases, such as dementia, anaemia, cardiovascular disease, neural tube defects, Crohn’s disease, celiac disease, and HIV. Vitamin B3 deficiency is linked to pellagra and cancer, while niacin (or nicotinic acid) lowers low-density lipoprotein (LDL) and triglycerides in the blood and increases high-density lipoprotein (HDL). A highly sensitive and robust liquid chromatography–tandem mass spectroscopy (LC/MS-MS) method was developed to detect and quantify a vitamin B3 vitamer (nicotinamide) and vitamin B6 vitamers (pyridoxial 5′-phosphate (PLP), pyridoxal hydrochloride (PL), pyridoxamine dihydrochloride (PM), pridoxamine-5′-phosphate (PMP), and pyridoxine hydrochloride (PN)) in human hair samples of the UAE population. Forty students’ volunteers took part in the study and donated their hair samples. The analytes were extracted and then separated using a reversed-phase Poroshell EC-C18 column, eluted using two mobile phases, and quantified using LC/MS-MS system. The method was validated in human hair using parameters such as linearity, intra- and inter-day accuracy, and precision and recovery. The method was then used to detect vitamin B3 and B6 vitamers in the human hair samples. Of all the vitamin B3 and B6 vitamers tested, only nicotinamide was detected and quantified in human hair. Of the 40 samples analysed, 12 were in the range 100–200 pg/mg, 15 in the range 200–500 pg/mg, 9 in the range of 500–4000 pg/mg. The LC/MS-MS method is effective, sensitive, and robust for the detection of vitamin B3 and its vitamer nicotinamide in human hair samples. This developed hair test can be used in clinical examination to complement blood and urine tests for the long-term deficiency, detection, and quantification of nicotinamide.

## 1. Introduction

Vitamin B and its vitamers (metabolites) are water-soluble nutritional elements critical for maintaining cellular metabolism, and cellular homeostasis, mainly as coenzymes. For example, nicotinamide (a vitamer of vitamin B3), pyridoxal 5′-phosphate (a vitamer of vitamin B6), pantothenic acid (vitamin B5), riboflavin (vitamin B2), biotin (vitamin B8), and 5-methyl tetrahydrofolate (a vitamer of vitamin B9) are all involved in neurotransmission and fatty acid synthesis, oxidation/reduction reactions, or one-carbon metabolism [1,2,3,4]. Vitamin B is usually obtained from foods such as almonds, whole grains, meat, fish, and leafy greens. However, a deficiency of vitamin B and its vitamers has negative effects on human health. Many diseases have been linked to the deficiency of vitamin B and its vitamers, such as cognitive impairment, anaemia [5,6], cardiovascular disease [7], neural tube defects [8,9], neuropsychiatric disorders [10], and thromboembolic processes [11].

**Figure 1:**
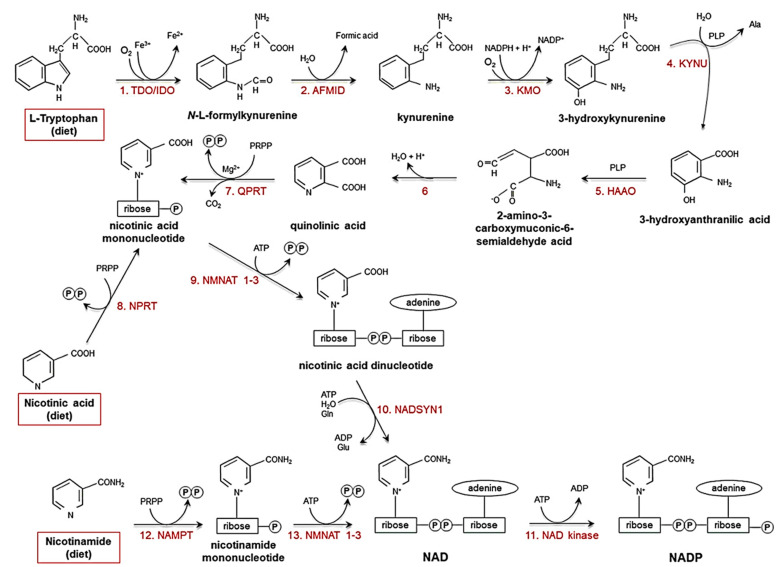
Pathways that biosynthesise NAD(P)H and niacin [16]. Ala: alanine; Glu: glutamate; Gln: glutamine; PRPP: 5-phosphoribosyl-1-pyrophosphate; PLP: pyridoxal phosphate. (Reproduced with permission from [16]).

Niacin, or nicotinic acid (a vitamer of vitamin B3), is absorbed by the body when dissolved in water and taken orally. It is converted to the major vitamer niacinamide in the body, along with other minor vitamers such as nicotinamide N-oxide and nicotinuric acid [12]. Niacin and nicotinamide are crucial for all living cells. Vitamin B3 is converted biosynthetically to nicotinamide adenine dinucleotide (NAD+) and nicotinamide adenine dinucleotide phosphate (NADP), oxidising agents that accept an electron from a reducing agent to change to the reduced form, NAD(P)H and are involved in DNA repair, calcium mobilisation, and deacetylation [12,13]. Moreover, NAD can be derived biosynthetically from the amino acid tryptophan through the kynurenine pathway. Niacin is also synthesised endogenously through the kynurenine pathway (see Figure 1) [14,15,16,17,18]. NAD+/NADH mediates redox reactions for energy metabolism and cellular biochemistry. NAD is also required as a coenzyme for the catalysis of oxidoreductases (dehydrogenases) [19]. Furthermore, NAD+/NADH is essential for mediating the electron transport chain, which fuels oxidative phosphorylation in mitochondria. Therefore, energy metabolism in cells is mainly mediated by cofactors derived from vitamin B3 and is involved in the majority of anabolic and catabolic pathways. Diseases caused by vitamin B3 deficiency are pellagra (dermatitis, depression, and diarrhoea) and cancer. Vitamin B3 (niacin form) is also known to lower total cholesterol, bad cholesterol (such as low-density lipoprotein (LDL)), triglycerides, and lipoprotein in the blood [12].

Vitamin B6, administered as pyridoxine hydrochloride, is used therapeutically to treat pyridoxine-responsive inherited disorders and some types of seizures in neonates and infants and to improve immune function in vitamin-B6-deficient individuals [20]. Pyridoxine has a hydroxymethyl group at four positions; pyridoxamine, an aminomethyl group; and pyridoxal, an aldehyde. Each vitamer may be phosphorylated at its 5-substituent to form an active coenzyme [21]. Pyridoxal 5′-phosphate (PLP) is the most common of these. It is the active form of vitamin B6 in humans and functions as a cofactor for more than 140 distinct enzyme-catalysed reactions [22].

Recently, many studies have been published on the analysis of vitamins B vitamers in human plasma, serum, and blood using LC/MS-MS. Asante et al. (2018) investigated the relationship between the deficiency of vitamin B vitamers involved in the folate-mediated one-carbon metabolism (FOCM) cycle and the pathogenesis of colorectal cancer, but this study was limited to plasma [23]. Zhang et al. (2019) examined the status of vitamins B1, B2, and B6 using dried blood spots (DBS) collected from Chinese children, but this assay was limited to only blood spots [24]. Roy et al. (2014) successfully quantified niacin and nicotinuric acid in humans, but the application was limited to human plasma [25]. Vitamin B3 vitamers, such as nicotinamide and nicotinuric acid, were also found in humans by Sutherland et al. but only in plasma [26]. Several studies have analysed nicotinamide in different matrices such as food, animal and human blood, serum, and plasma to quantify nicotinamide by LC/MS-MS, but there is no literature on the analysis and quantification of vitamin B vitamers in human hair using LC/MS-MS [27,28,29,30].

Zgaga et al. (2019) [31] and another study published by our lab recently [32] analysed and quantified 25-hydroxyvitamin D concentrations in human hair using LC/MS-MS. These are the first two studies to use human hair and beard samples for LC/MS-MS to quantify 25(OH)D3 and to show the existence of 25(OH)D3 in human hair samples. Zgaga et al. (2019) [31] compared the 25(OH)D3 levels in human serum and hair samples. Hair samples showed 25(OH)D3 hormone levels for an extended period, depending on the hair length, whereas serum samples showed short-lived 25(OH)D3 levels. Furthermore, there was a large and small variation in 25(OH)D3 levels between and within-subjects, respectively, as opposed to slightly similar concentrations in serum samples. Therefore, hair testing should be applied as a complementary test and not as a replacement for blood testing for 25(OH)D3 levels for clinical examination [31].

Hair samples could be easily collected (non-invasive), stored and transported without any fear of infection, while blood collection required trained professionals, and storage requires freezing at very low temperatures and special care. Hair analysis could be useful in verifying self-reported histories of nicotinamide deficiency. It has been well documented in literature that hair analysis has a wider window of detection and could show the presence of analytes in a person’s body from a few weeks up to a year, while blood and urine tests could detect the presence of analyte from 2–4 days. This could confirm the long-term deficiency of analyte or could show if the deficiency was more recent. Lastly the hair test could be used as a complementary test to blood and urinalysis as the studies revealed [31,33,34].

Given the advantages of hair testing, the objective of this study was to develop and validate an innovative LC/MS-MS method for the accurate detection and quantification of vitamin B3 and B6 vitamer in human hair samples collected from the UAE population. The secondary aim was to use the validated LC/MS-MS assay for accurate measurement of vitamin B3 and B6 vitamer levels in the hair samples collected.

## 2. Results

### 2.1. Development of an LC-MS/MS-Based Method

The method of extracting vitamin B3 and B6 vitamers, along with a robust and sensitive LC-MS/MS for vitamer analysis, was developed after investigating many extraction techniques. Next, we will discuss the series of experimental methods that were performed. First, we identified the product ions of each precursor ion of each analyte. Subsequently, the multiplier voltage (Delta EMV) was subjected to variation to check its effect on the peak intensity produced. The effect of different column types on the resolution of the peaks obtained was examined. Lastly, the method reproducibility was tested and tried, in addition to analysing the recovery of all the analytes. Based on the in-depth study of the parameters mentioned above, the method optimisation state was reached and evaluated.

### 2.2. Multiple Reaction Monitoring (MRM) (Precursors and Product Ion Identification)

Compounds with different structures behave differently to various fragmentor voltage settings. The injection of individual standards enabled the detection of the ideal fragmentation voltage for every single component. The fragmentor was set to positive electrospray ionisation mode. The positively charged molecules of analytes entered the mass spectrometer. Precursor ions were generated, which, by collision-induced dissociation, formed product ions. Both precursor and product ions were measured together in multiple reaction monitoring (MRM) mode. The daughter ions were found based on the mass of the standards at varying collision energies. The values of nicotinamide, pyridoxamine 5′-phosphate (PMP), pyridoxamine (PM) [35], and pyridoxal 5′-phosphate (PLP) [17] matched those in the literature, while the values of pyridoxal (PL) and pyridoxine (PN) were close to those in the literature [35].

PLP showed a precursor ion of *m*/*z* 248 with complete fragmentation and produced an intensity signal of the quantifier product ion of *m*/*z* 149.7 and qualifier product ion of 94 with collision energies 15 eV. The precursor ion of *m*/*z* 168 for PL was completely fragmented and produced an intensity signal of the quantifier product ion of *m*/*z* 149.9 and qualifier product ion of 94 with collision energies 10 eV. The PMP quantifier product ion of *m*/*z* 232.1 and qualifier ion of 134.1 was detected at collision energies 10 eV. PM showed a quantifier product ion of *m*/*z* 152 and qualifier product ion of 134.1 at collision energies 10 eV, produced from the intense precursor ion of *m*/*z* 169. The quantifier product ion of *m*/*z* 151.9 of PM was the strongest at collision energy 10 eV and it was used for quantitation while the qualifier product ion was 134.1 at 10 eV collision energy. Nicotinamide had a fully fragmented precursor ion of *m*/*z* 123 with a high-intensity quantifier product ion of *m*/*z* 80.2 and a qualifier product ion of 96 at collision energy 20 eV. The internal standard showed a daughter quantifier ion at 155.1 and a qualifier ion at *m*/*z* 137.1 at collision energy 10 eV. Table 1 summarises the precursor and product ion, fragmentor voltage, and collision energy values found for every analyte.

Two columns with distinct dimensions and similar packing materials were used to test the chromatographic separation method. Eclipse plus C-18 was first column tested with a 1.8 µm particle size, 50 mm length, and inner diameter 2.1 mm, while the second column was Poroshell 120 EC-C18 with a 2.7 µm particle size, 100 mm length, and 3.0 mm inner diameter. The investigation was based on testing which column would give the highest resolution and intensity. Both columns showed an almost similar number of peaks, but differences were observed in elution profiles. However, the Poroshell column obtained a chromatographic profile with a separated peak with high intensity compared with the Eclipse plus C-18 column, whose chromatographic profile showed separated, broad peaks [36].

**Figure 2:**
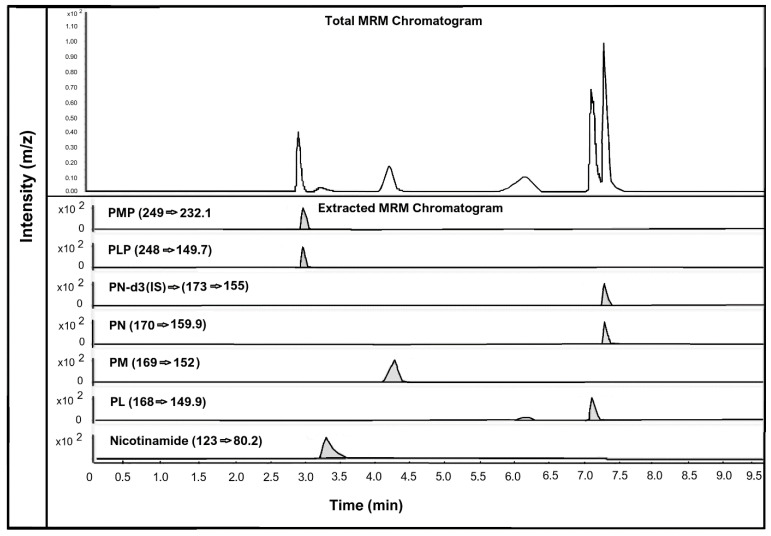
LC-MS chromatogram of all vitamin B3 and vitamin B6 vitamers analysed in human hair. All analytes spiked at a concentration of 150 pg/mg of hair.

The human hair samples were analysed for estimating and detecting seven different vitamin B3 and B6 vitamers: nicotinamide (vitamin B3 vitamer); PMP, PM, PLP, PN, and PL (vitamin B6 vitamers); and the IS. However, only nicotinamide was detected and quantified in the human hair samples. This suggests that nicotinamide is the only vitamin B3 form that can be quantified in human hair. Figure 2 below shows the chromatographic peaks and MRM transitions of vitamin B3 vitamer, nicotinamide, and vitamin B6 vitamers PMP, PM, PLP, PN, and PL along with the internal standard PN-d3.

**Figure 3:**
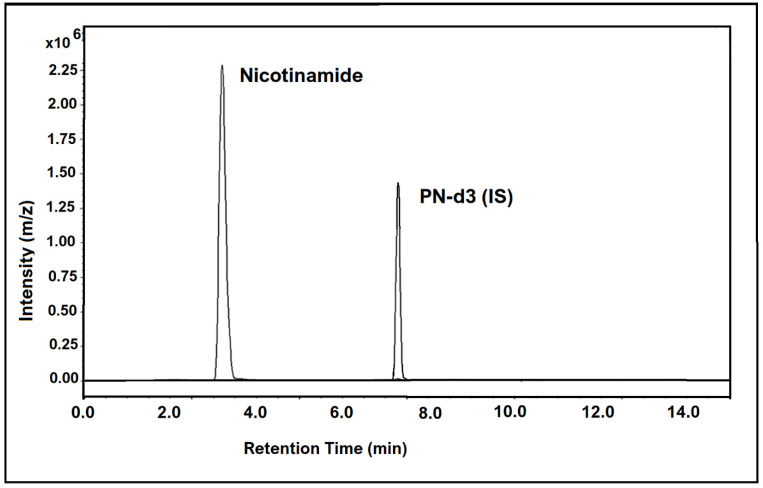
LC-MS/MS chromatogram showing nicotinamide (vitamin B3 vitamer) found in human hair at a concentration of 758.8 pg/mg (sample 27), along with the internal standard peak.

The chromatogram in Figure 3 below shows the chromatographic peaks and MRM transitions for nicotinamide in human hair samples, along with the internal standard.

## 3. Method Validation

The LC-MS/MS validation method was evaluated using the following parameters: linear range, the intra- and inter-day accuracy and precision, recovery LOD and LOQ. The validation results are presented in Table 2. Table 2 summarises the intra- and inter-day precision and accuracy of three QC samples. The intra-day precision and accuracy values were all within the acceptable range. The percentage recovery of the low, medium, and high QC of vitamin B3 and B6 vitamers is also given in the Table 2 below. The recovery ranged between 73% and 89%. The LOD was 10 pg/mg, and LOQ was 50 pg/mg with a linear range from 50 to 4000 pg/mg.

### Analysis of Human Hair Samples

The analysis results for the 40 hair samples collected are given in Table A1. The nicotinamide concentration was 104.9 to 2706.5 pg/mg of hair in the female participants and 106.9 to 3349.9 pg/mg of hair in the male participants.

**Figure 4:**
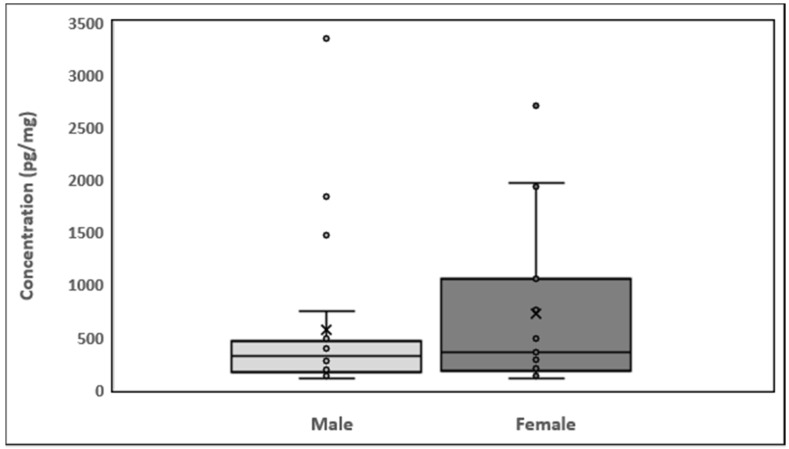
Box plots showing the distribution of nicotinamide in hair and data skewness through data quartiles, “**ˣ**” shows the mean nicotinamide concentrations and “•” shows the outliers.

**Figure A1:**
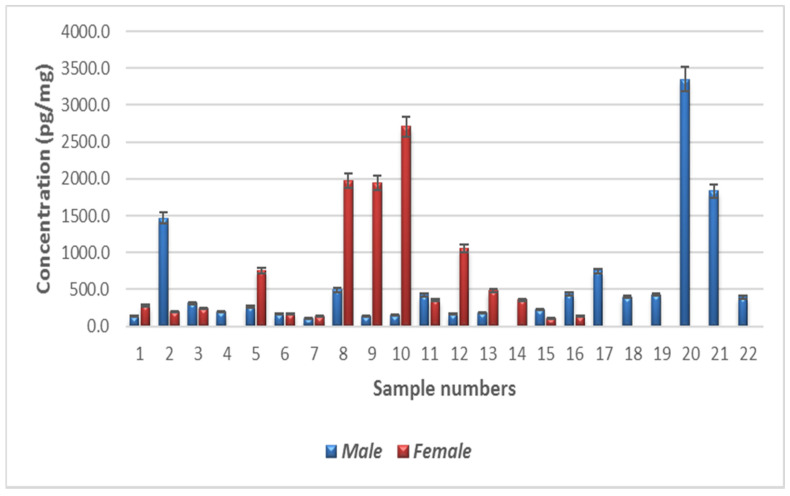
Levels of nicotinamide in the human hair samples. Error bars above shows the standard error of the mean.

The minimum nicotinamide concentration was 106.9 pg/mg in males and 104.9 pg/mg in females (Figure 4). The maximum nicotinamide concentration was 3349.9 pg/mg in males (sample 14) and 2706.5 pg/mg in females (sample 4). Quartile 1 values for male and female participants were 171.5 and 187.1 pg/mg, respectively, and quartile 3 values were 443.4 and 905.9 pg/mg, respectively. Mean nicotinamide concentrations for male and female participants were 573.7 and 726.6 pg/mg, respectively. The interquartile range (IQR) was −236.4 to 851.42 pg/mg. The average nicotinamide concentrations in male and female participants are shown in Table A1. In Figure A1, the results are represented as a histogram detailing the nicotinamide ranges for the male and female participants. The graph depicts the sample numbers vs. the nicotinamide concentrations quantified in each sample.

## 4. Discussion

Nicotinamide was found in all hair samples, except one male and three female samples that had no nicotinamide. The exact reason behind these results is unknown. The results between the two genders were compared, and no major differences were found. These findings prove that nicotinamide, a vitamin B3 vitamer, can be easily detected in human hair. Further research is required to detect other vitamin B vitamers compared to blood and urine tests. The results also show the prevalence and distribution of nicotinamide in hair. Of 40 samples, 12 were in the range 100–200 pg/mg, 15 in the range of 200 to 500 pg/mg, 9 in the range of 500–4000 pg/mg, and 4 samples with no nicotinamide.

In this study, we detected nicotinamide, a vitamin B3 vitamer, in human hair. As there are no previous studies on vitamin B vitamers and their levels in human hair, we have just correlated our results with what is available in the literature: the nicotinamide quantified in human hair samples in our study was compared with the average levels of nicotinamide quantified in human plasma. Redeuil et al. (2015) quantified 21 water-soluble vitamin Bs in human plasma samples of the American population. Since the only vitamin B3 vitamer found and quantified in human hair samples was nicotinamide, we wanted to find the average levels of nicotinamide in human blood samples and it seemed rational to compare it with the results of Redeuil et al. [35]. A comparison of our human hair results of average nicotinamide (104.9 to 3349.9 pg/mg) with average levels of nicotinamide in human plasma (69.1–479.6 nmol/L, or 8.5–59 ng/mL) shows good correlation [35].

Hair testing is also non-invasive compared with blood testing. Hair samples can be easily collected and transported, with no fear of infection. Moreover, hair is easily storable, whereas blood, urine, and saliva storage requires special care [37]. Hair testing has also demonstrated that the concentration of parent drug ions is higher than product ions compared with urinalysis [37]. Segmental hair analysis can confirm nicotinamide deficiency information of the past few weeks to many months, depending on the length of the hair analysed, while in urine and blood analytes, nicotinamide can be detected up to 2 to 4 days [33,34]. The practical major advantage of hair analysis over blood and urine tests for nicotinamide deficiency is its wider window of detection. The presence of a vitamin or its vitamer in hair can confirm whether the person has a long-term deficiency of nicotinamide or has become recently deficient due to a disease or dietary constraints. In addition, hair analysis is useful when a history of nicotinamide deficiency is difficult to obtain (e.g., in psychiatric patients). Due to these characteristics, hair analysis is extremely valuable, especially when the other biological matrices are unsuitable. In hair analysis, however, the interpretation of results is still debatable because of some unanswered questions, such as the effect of cosmetic treatment, genetic differences, and external contamination [38]. In addition, the hair matrix should not be considered an ultimate substitute of urine and/or blood matrices in evaluating analyte deficiency but rather as a source of important and complementary information. Due to these benefits, hair testing will continue to develop further, and new applications will be discovered with time.

The nicotinamide concentrations in hair are always low as a small amount of hair is analysed and a small amount of nicotinamide usually reaches and gets stored in the hair shaft. This also depends on the biochemical composition of the hair and blood cells, interaction with other cells, lifestyle, polymorphism, and diet. The next step is to compare the human blood and hair vitamin B3 levels and also perform a segmental analysis of hair samples to depict the long-term deficiency of nicotinamide and other vitamin B vitamers in individuals, which will be a good predictor of obesity and high cholesterol and many other diseases.

## 5. Materials and Methods

### 5.1. Sample Collection

For this study, 40 healthy male and female students (aged 18–35 years) from the UAE University were recruited in December 2019. All the participants read the participant information sheet and signed consent forms as per the UAE University ethical approval protocol (UAEU Ref# SNA/fa/19-15). All participants were in good health and free from any known diseases and vitamin deficiencies. About 500 mg of hair was collected from the crown. A rubber band was used to tie hair strands. Hairs were cut close to the surface of skin and collected from different spots on the crown to avoid creating a bald spot. The samples were stored in labeled plastic envelopes in a cold, dry, and dark place before processing. The length of the hair samples was approx. 6 cm. Six different vitamin B3 (nicotinamide) and B6 vitamers (pyridoxial 5′-phosphate (PLP), pyridoxal hydrochloride (PL), pyridoxamine dihydrochloride (PM), pridoxamine-5′-phosphate (PMP), and pyridoxine hydrochloride (PN)) were analysed in the hair samples; pyridoxine-(methyl-d3) hydrochloride was used as the internal standard.

### 5.2. Chemical and Reagents

PN, PMP, PM, PLP, PL, nicotinamide, heptafluorobutyric acid, and LC-MS-grade water were purchased from Sigma-Aldrich, St. Louis, MO, USA. Formic acid, isopropanol, methanol, ethyl acetate, acetonitrile, deionised water, ammonium hydroxide, ammonium formate, and pentane were obtained from LABCO LLC (Dubai, UAE).

### 5.3. Preparation of Standard Solutions

Individual stock solutions of all the vitamin B3 and B6 vitamers and the internal standard were prepared at a concentration of 1 ppm (1000 µg/mL) in formic acid solution. Stock solution standards were mixed to form a working solution with a specific composition. Calibration was performed by the working solution. The stock solutions were used to prepare quality control (QC) samples. Each stock solution was diluted with DI water to the desired concentration to prepare working solutions of vitamin standards and internal standard. The solutions were prepared using amber glass tubes to prevent the degradation of vitamins by the light of the laboratory. Standard mixture solutions were prepared by diluting individual stock solutions. Hair powder containing no vitamin B3 and B6 vitamers were spiked with different concentrations to prepare calibrants and quality control samples. All solutions were stored at −20 °C in amber glass tubes until further processing.

### 5.4. Extraction Method

The 500 mg hair samples were decontaminated with isopropanol to remove contaminants, sweat, sebum, and coloring. The hair strands were dried under a gentle stream of air. The decontaminated hair was ground into a fine powder using a Fritsch Mini-ball mill (Fritsch and Gerhardt UK Ltd., Brackley, UK). Internal standard solution was added to all samples calibrants and QC’s except blank hair sample. The powder was transferred into a test tube, 2 mL of methanol was added, and the mixture was vortexed for a few minutes. The mixture was further subjected to sonication for 60 min in a water bath at 35 °C to release hair-bound vitamin B3 and B6 vitamers from the hair matrix. For extraction, we tried water alone and then a water/methanol mixture, but extraction with methanol was found to give the best release. The hair mixture was centrifuged at 1500× *g* for 5 min to obtain the supernatant (expected vitamin B3 and B6 vitamers). The supernatant was collected and filtered through a syringe filter having a PTFE membrane (0.45 μm). The filtrate was dried in a sample concentrator using a gentle stream of N_2_ at 40 °C and reconstituted with 50 µL of a methanol:water (50:50, *v*/*v*) mixture, and 20 µL was injected into the LC-MS/MS system.

### 5.5. Liquid Chromatography–Tandem Mass Spectrometry (LC-MS/MS)

The separation and quantification of analytes were performed using 8060-LCMS coupled with Nexera ultra-high-pressure liquid chromatography (UHPLC), Shimadzu Corporation (Kyoto, Japan). The quantification of each analyte was performed using the signature multiple reaction monitoring (MRM). The UHPLC system is equipped with a reversed-phase Poroshell EC-C18 column with a particle size of 2.7 µm, a length of 100 mm, and a diameter 3.0 mm. The column was kept at 35 °C with a constant flow rate of 0.4 mL/min. The elution of analytes was carried out with two mobile phases: A comprising LC-MS-grade water consisting of 0.1% formic acid and 0.1% heptafluorobutyric acid and B comprising acetonitrile with 0.1% formic acid. LC started with 100% A for 3 min, followed by 0–100% B for 3–5 min, then 100% B from 5–8.50 min, and declined to 100% A from 8.60–10 min. Then, the column was recovered to the initial conditions in the post-run for 5 min by eluting with 100% of the mobile phase. Heptafluorobutyric acid has been used and added to the mobile phase in the past for optimal retention and separation, as an ion-pair reagent in mass spectrometry. It has also been used in the analysis of highly polar compounds and for robust and sensitive detection of metabolites related to the tryptophan-kynurenine pathway and quantitative profiling of biomarkers related to B-vitamin status [39].

For the optimisation of the parameters for LC-MS/MS, first, a series of steps was performed to liberate the analytes from the hair. Then, a suitable separation method was performed, which gave well-separated peaks with high resolution. As soon as the optimised conditions were determined, they were used to investigate analyte recovery, similar to the analysis of spiked human plasma samples performed in our laboratory previously [36].

A Shimadzu 8060 Triple Quadrupole MS system in positive electrospray ionisation (ESI+) mode was used to perform mass spectroscopy analysis. The electrospray voltage was set at 4000 V, and the optimal conditions were follows: ion source gas (1) set at 20 psi, ion source (2) set at 45 psi, and the flow of the drying gas (N_2_) set at 8 L/min at a source temperature of 300 °C. The names, structures, and molecular masses of B6 and B3 vitamers are shown in Table 3 below.

### 5.6. Method Validation

The LC-MS/MS method was validated by examining the following parameters: linear range, lower limit of detection (LOD), lower limit of quantitation (LOQ), intra- and inter-day accuracy and precision and recovery. The US Food and Drug Administration (US-FDA) guidelines were used for bioanalytical method development and validation [40]. Intra- and inter-day accuracy and precision were determined for three quality control (QC) samples. The evaluation of intra- and inter-day accuracy and precision was performed at high, low, and medium QC sample concentrations by investigating the replicates at every level. The inter-day precision accuracy was accepted if the experimental concentrations were within 15% of the actual concentrations and the lower limit of quantification (LOQ) was accepted within the 20% limit range.

The inter/intra-day precision (% *CV*) data were calculated from quality control data obtained after analysis using the equation:

(1) $$\left[\%\;CV=\left(\frac{Standard\;deviation}{mean}\right)\times 100\right]$$

The assay % inter/intra-day accuracy was obtained from the quality control data using the equation:

(2) $$\left[\%\;Accuracy=\left(\frac{Mean\;value}{Nominal\;value}\right)\times 100\right]$$

The % absolute recoveries were calculated using the equation:

(3) $$\left[\%\;Recovery=\left(\frac{Mean\;unextracted\;QC\;values}{Mean\;extracted\;QC\;values}\right)\times 100\right]$$

The lower limit of detection (LOD) was established by comparing the instrument signal to noise ratio (S/N) to the analytes lowest concentration. The analytes lowest concentrations were determined by decreasing the analyte concentrations until an LC-MS/MS detector response equal to 3 times the instrument background noise level was observed.

Quality control samples were prepared at the following concentrations: QCH at 1200 pg/mg, QCM at 400 pg/mg, and QCL at 200 pg/mg. All QC and calibrant samples were prepared with human hair containing no vitamin B3 and B6 vitamers or no interfering or matrix peaks at required retention times. For analyte recovery, QC samples at the above three concentrations were prepared in methanol, dried, reconstituted in the mobile phase, and injected into the LC-MS/MS system and the area under the curve for all QC samples was calculated. Then, QC samples at the same concentrations were spiked in blank human hair samples, extracted using the normal protocol, dried, reconstituted, and injected into the LC-MS/MS system, and the area under the curve was then calculated. The percentage recovery was calculated by taking the ratio of extracted vs. non-extracted vitamin B3 and B6 vitamers and multiplying it with 100. The specificity of the method was determined by running 6 blank hair samples and finding no interfering peaks at the retention times of vitamin B3 and B6 vitamers.

From the 40 human hair samples, six different lots of presumed vitamin B3- and B6-free samples were ball-milled, and one representative sample from each lot was selected. The selected samples were extracted and analysed in triplicate using LC-MS/MS. No interferences from the matrix and no vitamer peaks were detected in few of the selected samples. One of the six samples was chosen as a blank; this sample was the cleanest of all, with no co-eluting and interfering peaks detected at retention time of the hair matrix and no vitamin B vitamer peaks. This blank hair sample was used for further preparation of quality controls and calibrants. Lastly, under the given analytical conditions employed, there were no major matrix interferences associated with blank hair that affected the analysis of vitamin B vitamers.”

## 6. Conclusions

To the best of our knowledge, this is the first pilot study to detect vitamin B3 and B6 vitamers in human hair. Our sensitive, rapid, and robust LC-MS/MS-based assay was able to quantify seven different vitamin B3 and B6 vitamers in human hair. Further work is warranted to develop and validate the procedure by including more vitamin B vitamers and to produce a gold-standard test for human hair analyses. This might be possible by using advanced sample concentration steps, using larger hair samples, and exploring more advanced sensitive commercially available instrumentation. Future investigative work should also include a full spectrum of ethnicity-based hair types, different population groups, age groups, genders, seasonal variations, and color combinations. Hair from other parts of the human body and animals could also be investigated. This innovation will help explore many serious diseases related to vitamin B deficiency.

In this study, human hair was chosen for the detection and quantification of vitamin B3 and vitamin B6 vitamers because hair is tamper-resistant and easily storable, with no risk of infection, and the method is non-invasive as opposed to human plasma testing. The method is free from interferences resulting from instrumental means and matrices. The LC-MS/MS method was validated with multiple parameters: linearity, reliability, robustness, specificity, rapidness, and accuracy. It will be possible to detect vitamin B3 coenzymes in human hair and prevent serious diseases. Therefore, the novel LC-MS/MS method is significant for future clinical trials and long-term evaluation of the vitamin B3 status in humans.

## Figures and Tables

**Table 1:** Fragmentation parameters identified for all analytes examined in this study. Transitions marked with * were used for quantitation.

Analyte	Precursor Ion (*m*/*z*) [M + H]^+^	Product Ion (*m*/*z*)	Fragmentor Voltage (V)	Collision Energy (eV)
PLP (Quantifier)	248 *	149.7	45	15
(Qualifier)	248	94	62	15
PL	168 *	149.9	94	10
	168	94	98	10
PMP	249 *	232.1	45	10
	249	134.1	60	10
PM	169 *	152	94	10
	169	134.1	98	10
PN	170 *	151.9	94	10
	170	134.1	97	10
Nicotinamide	123 *	80.2	94	20
	123	96	90	20
PN-d3	173 *	155	94	10
	173	137.1	90	10

**Table 2:** LC-MS/MS validation parameters for intra- and inter-day accuracy, precision, and recovery.

Analytes	Conc. QC’s (pg/mg)	% Recovery	Intraday (*n* = 6)		Interday (*n* = 6)	
			Precision, % CV	Accuracy, %	Precision, % CV	Accuracy, %
Nicotinamide	200	73	11.6	94.2	9.2	91.1
	400	78	6.3	88.2	11.5	86.5
	1200	87	7.5	87.0	8.5	85.6
PLP	200	81	12.5	88.9	12.2	89.8
	400	89	14.3	89.4	13.3	88.9
	1200	91	12.9	89.9	12.3	87.8
PL	200	85	13.5	112.2	14.7	94.5
	400	86	9.1	102.3	10.9	91.6
	1200	78	5.3	100.1	8.9	93.9
PMP	200	86	14.9	92.8	13.2	100.5
	400	82	13.9	85.6	12.7	86.9
	1200	89	7.2	89.9	13.2	94.3
PM	200	78	11.2	98.3	9.3	85.2
	400	89	8.3	102.6	8.1	87.3
	1200	81	6.1	100.9	13.6	87.1
PN	200	82	13.8	93.3	4.3	101.2
	400	84	7.5	98.7	12.1	89.8
	1200	87	8.2	101.2	13.2	99.9

**Table 3:** Names, structures, and masses of B6 and B3 vitamers.

Name	Vitamer Structure	Molecular Mass (g/mol)
**Pyridoxal-5’-phosphate** **(PLP) (vitamin B6 vitamer)**	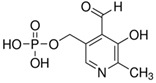 **C_8_H_10_NO_6_P**	247.1
**Pyridoxal hydrochloride** **(PL) (vitamin B6 vitamer)**	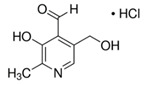 **C_8_H_9_NO_3_ · HCl**	203.6 167.1 (− Cl)
**Pyridoxamine dihydrochloride** **(PM) (vitamin B6 vitamer)**	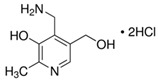 **C_8_H_12_N_2_O_2_ · 2HCl**	241.1 168.1 (− 2HCl)
**Pyridoxamine-5’-phosphate** **(PMP) (vitamin B6 vitamer)**	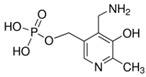 **C_8_H_13_N_2_O_5_P**	248.1
**Pyridoxine hidrochloride** **(PN) (vitamin B6 vitamer)**	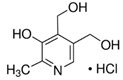 **C_8_H_11_NO_3_ · HCl**	205.6 169.1 (− HCl)
**Nicotinamide (vitamin B3 vitamer)**	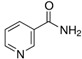 **C_6_H_6_N_2_O**	122.1
**Pyridoxine-(methyl-d3) hydrochloride**	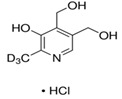 **C_8_H_9_NO_3_ · HCl**	208.6

## Data Availability

All relevant data are included in the paper or its Supplementary Information.

## Sample Availability

Samples of the compounds are not available from the authors.

## Appendix A

**Table A1:** The average concentrations of nicotinamide in male and female volunteers. (here, SD means standard deviation and “ND” means “not detected”).

Male		Female	
Hair Sample	Mean ± SD (pg/mg)	Hair Sample	Mean ± SD (pg/mg)
Sample-01	131.1 ± 6.72	Sample-23	284.4 ± 7.11
Sample-02	1468.4 ± 3.9	Sample-24	200.8 ± 3.1
Sample-03	316.8 ± 14.2	Sample-25	243.1 ± 2.1
Sample-04	195.6 ± 0.62	Sample-26	ND
Sample-05	269.6 ± 3.29	Sample-27	758.8 ± 1.49
Sample-06	169.2 ± 17.2	Sample-28	173.3 ± 2.5
Sample-07	107.0 ± 2.3	Sample-29	132.1 ± 4.2
Sample-08	493.1 ± 3.23	Sample-30	1972.1 ± 1.1
Sample-09	135.6 ± 8.2	Sample-31	1939.5 ± 3.1
Sample-10	147.7 ± 1.59	Sample-32	2706.0 ± 4.4
Sample-11	422.1 ± 5.62	Sample-33	356.1 ± 0.5
Sample-12	171.5 ± 5.11	Sample-34	1052.9 ± 0.9
Sample-13	186.7 ± 9.93	Sample-35	483.7 ± 3.1
Sample-14	ND	Sample-36	358.5 ± 8.1
Sample-15	224.9 ± 11.2	Sample-37	105.0 ± 0.9
Sample-16	443.4 ± 4.7	Sample-38	131.6 ± 3.2
Sample-17	747.5 ± 3.9	Sample-39	ND
Sample-18	400.2 ± 2.1	Sample-40	ND
Sample-19	430.8 ± 3.5		
Sample-20	3350 ± 0.71		
Sample-21	1837.1 ± 1.2		
Sample-22	397.9 ± 1.1		
